# Predictors of infusion reactions during infliximab treatment in patients with arthritis

**DOI:** 10.1186/ar2020

**Published:** 2006-07-26

**Authors:** Meliha C Kapetanovic, Lotta Larsson, Lennart Truedsson, Gunnar Sturfelt, Tore Saxne, Pierre Geborek

**Affiliations:** 1 Department of Rheumatology, Lund University Hospital, Lund, Sweden; 2 Department of Clinical Microbiology and Immunology, Lund University Hospital, Lund, Sweden

## Abstract

In the present study we evaluated the impact of baseline antinuclear antibody (ANA) status and use of methotrexate on development of infliximab-related infusion reactions in patients with rheumatoid arthritis (RA) or spondylarthropathies (SpAs), including psoriatic arthritis. All patients with RA (*n *= 213) or SpA (*n *= 76) treated with infliximab during the period 1999–2005 at the Department of Rheumatology in Lund, Sweden were included. ANAs were present in 28% and 25% of RA and SpA patients, respectively. Because of differences in baseline characteristics, we used a binary logistic regression model to calculate odds ratios (ORs), adjusting for age, sex and prednisolone dosage. Altogether 21% of patients with RA and 13% of patients with SpA developed infusion reactions (*P *= 0.126). The OR for development of infusion reactions in RA patients with baseline ANA positivity alone was 2.1. Infliximab without methotrexate and infliximab as monotherapy were associated with ORs of 3.1 and 3.6, respectively. Combining infliximab without methotrexate and ANA positivity yielded an OR for infusion reaction of 4.6. Lower age at disease onset and longer disease duration were associated with infusion reactions (*P *= 0.012 and *P *= 0.036, respectively), but age, sex, C-reactive protein, erythrocyte sedimentation rate, Health Assessment Questionnaire and Disease Activity Score-28 at baseline were not. No predictors of infusions reactions were identified in SpA patients. RA patients treated with infliximab without methotrexate, and who are positive at baseline for ANAs are at increased risk for developing infliximab-related infusion reactions.

## Introduction

Treatment with infliximab, a chimeric IgG_1 _antibody that is specific for human tumour necrosis factor-α, has been shown to be effective in treating a variety of inflammatory diseases. In combination with methotrexate, infliximab provides significant and sustained improvement in a majority of patients with rheumatoid arthritis (RA) [1,2] but also in spondylarthropathies (SpAs), including psoriatic arthritis [3,4]. However, one of the clinical problems associated with infliximab treatment is development of infusion reactions. Acute infusion reactions occur within 24 hours and delayed ones develop 2–14 days after initiation of treatment. Acute reactions can be true allergic, namely IgE-mediated type I reactions (anaphylactic reactions), including hypotension, bronchospasm, wheezing and/or urticaria. However, the great majority of infusion reactions reported during infliximab treatment are characterized by more nonspecific symptoms and are often classified as anaphylactoid ones (i.e. probably nonallergic) [5]. A range of symptoms including headache, nausea, fever or chills, dizziness, flush, pruritus, and chest or back pain have been described in relation to infusions, but these do not necessarily require discontinuation of treatment [1,2,5].

It has been shown that infliximab treatment can induce development of antidrug antibodies that lead to infusion reactions and mandate withdrawal of treatment [1,2]. Maini and coworkers [2] observed that low-dose methotrexate added to infliximab reduced the development of antidrug antibodies in groups of patients, suggesting that addition of methotrexate could possibly reduce immunogenicity against the monoclonal antibodies. Also, concomitant treatment with different immunosuppressive agents in patients with Crohn's disease has been shown to reduce the incidence of infusion reactions [6].

In addition to development of anti-infliximab antibodies, induction of different autoantibodies, including antinuclear antibodies (ANAs), has been described during infliximab treatment in both RA and SpA patients [7-10]. With respect to ANAs, new appearances but also shifts in ANA status have been detected in RA patients during treatment with disease-modifying antirheumatic drugs (DMARDs) [11]. Also, treatment with tumour necrosis factor blockers has been shown to lead not only to induction of ANAs but also to a switch from ANA positivity to ANA negativity [7-10]. The clinical significance of new appearance of ANAs has been addressed in several studies [7-11]. Cases of lupus-like syndrome have been reported, but in the majority of patients the appearance of ANAs did not have any clinical significance [7,8]. Furthermore, a correlation between ANA positivity and toxic effects of drugs (i.e. some DMARDs) was previously reported [12,13]. Toxic reactions to gold compounds and penicillamine were also found to be more prevalent among RA patients with certain HLA-DR alloantigens [14], but there are insufficient data on the impact of ANA status at baseline on risk for development of infusion reactions in relation to infliximab treatment.

A pilot study including patients with RA [oral presentation at Meeting of the Swedish Rheumatology Society 2003, unpublished data] showed that positive baseline ANA was a risk factor for developing infusion reactions, particularly when infliximab was used as monotherapy. The aim of the present study was to evaluate the predictive value of ANA status, methotrexate and other concomitant immunomodulating agents before the initiation of infliximab treatment for development of infusion reactions during infliximab treatment in patients with chronic arthritis treated clinically.

## Materials and methods

### Patients

The study population consisted of patients with RA (*n *= 213) or SpA (*n *= 76) treated with infliximab during the period 1999–2005 at the Department of Rheumatology in Lund, Sweden. In order to ensure that all RA patients fulfilled American College of Rheumatology (ACR) 1987 criteria [15], a systemic review of medical records was performed. The SpA group included 21 patients fulfilling 1984 New York revised classification criteria for ankylosing spondylitis [16], 43 patients with psoriatic arthritis according to the classification criteria proposed by Moll and Wright in 1973 [17], five patients with inflammatory bowel-related arthritis, and seven patients with undifferentiated SpA. All patients were included in the South Swedish Arthritis Treatment Group protocol (SSATG) follow-up system for monitoring of treatments with biologics [18]. The evaluations included swollen and tender joint counts, assessment of pain (visual-analogue scale), patient overall assessment (visual-analogue scale), physician's global assessment (five grades), and concomitant treatment with DMARDs and oral glucocorticoids. Disease Activity Score-28 was calculated for RA patients and used to grade disease activity [19]. Infliximab was given to both patient groups at a dosage of 3 mg/kg at the start of treatment, after 2 and 6 weeks, and as a rule every 8th week thereafter, as recommended by the manufacturer. The dosage could be increased or treatment intervals shortened in case of insufficient clinical response to treatment. Clinical evaluations and blood sample collection were performed directly before infusions.

All adverse events, including infusion reactions, were registered and seriousness graded by one investigator. Grades of seriousness were as follows: mild, moderate, serious and life threatening. Mild reactions were defined as self-limiting and resolving after temporary stop/slowing of infusion. Moderate reactions were those that required closer attention, an extended observation period and often a stop to the infusion. Serious reactions involved a infusion, respiratory symptoms/symptomatic blood pressure fall and need for close monitoring, often for a whole day and occasionally requiring ward referral. Life-threatening reactions were those that required intensive care treatment. An infusion reaction was defined as an adverse event occurring during infusion or within 24 hours after initiation of infusion.

### Determination of antinuclear antibody status

ANA status was analyzed at initiation of infliximab treatment. In case of missing data, ANA status within a month before treatment start was used. The measurement of ANAs was performed using indirect immunofluorescence assay with HEp2 cells as substrate and anti-IgG conjugates, as described previously [20]. The analysis was conducted using an accredited method at the Department of Clinical Microbiology and Immunology, Lund University Hospital, Lund, Sweden (accredited according to SS-EN ISO/IEC 17025). Values 14 units/ml or greater, corresponding to a titre of 400, were considered positive. The reference interval was based on results of measurements in healthy blood donor control individuals, and the upper limit was determined to result in between 1% and 5% of control individuals being positive for ANAs.

### Statistical analysis

Statistical analysis and calculations were performed using SPSS 13.00 software (SPSS Institute Inc., Cary, NC, USA). Because of differences in baseline characteristics, predictive values were determined using a binary logistic regression model adjusting for age, sex and prednisolone dosage. The impact of continuous variables was estimated using the Mann-Whitney U-test. Differences in infusion reactions between RA and SpA patients were analyzed using Fisher's exact test. *P *< 0.05 was considered statistically significant.

## Results

Altogether, 213 RA patients and 76 SpA patients were treated with infliximab during the period 1999–2005. Demographics, disease characteristics, treatment characteristics and disease activity variables are summarized in Tables 1 and 2. The proportion of women was greater in RA than in SpA patients. RA patients were older at initiation of infliximab treatment but disease duration at inclusion did not differ between RA and SpA patients. Age at disease onset was lower in SpA patients. Baseline ANA status did not differ significantly between RA and SpA patients, and there were missing data in only 12 (5.6%) RA and four (5.3%) SpA patients. Also, mean Health Assessment Questionairre, C-reactive protein and erythrocyte sedimentation rate differed between the two patient groups.

Infliximab was given as monotherapy (i.e. without other DMARDs) in 46 (21.6%) RA and 31 (40.8%) SpA patients. Among concomitant DMARDs, methotrexate was most frequently prescribed in both patient groups, although a larger proportion of RA (60.6%) than of SpA (46.1%) patients received methotrexate. The methotrexate dosage did not differ between the groups at the start of treatment compared with when the infusion reaction occurred. Sulphasalazine, azathioprine and other DMARDs were used less frequently in both patient groups. The mean number of previous DMARDs was 3.3 (range 1–9) and 1.9 (range 0–5) in RA and SpA groups, respectively. A substantial proportion of RA patients were receiving concomitant prednisolone treatment at baseline compared with SpA patients.

A larger proportion of RA patients (21.1%) than of SpA patients (13.2%) developed some form of infusion reaction during the treatment, but this difference failed to reach statistical significance (Fischer's exact test; *P *= 0.126). The treatment duration before the infusion reaction occurred was significantly shorter in SpA patients than in RA patients (Mann-Whitney U-test; *P *= 0.006).

The infliximab dosage was increased in 21 out of 45 (46.7%) RA patients and in three of 10 (30%) SpA patients who developed infusion reactions. Among RA patients the dosage was increased at between 3 and 6 months of treatment duration in 11 patients, at between 6 and 12 months in seven patients, and after 12 months in nine patients. The corresponding numbers of SpA patients were 3, 2 and 0. The infliximab dosage was increased more than once in six RA and two SpA patients. No significant correlation between increased dosage over time and development of infusion reactions was found (χ^2 ^test). For comparison there were increases in dosage in 98 out of 168 (58.3%) RA patients and in 38 out of 66 (57.6%) SpA patients who did not develop an infusion reaction.

**Figure 1:**
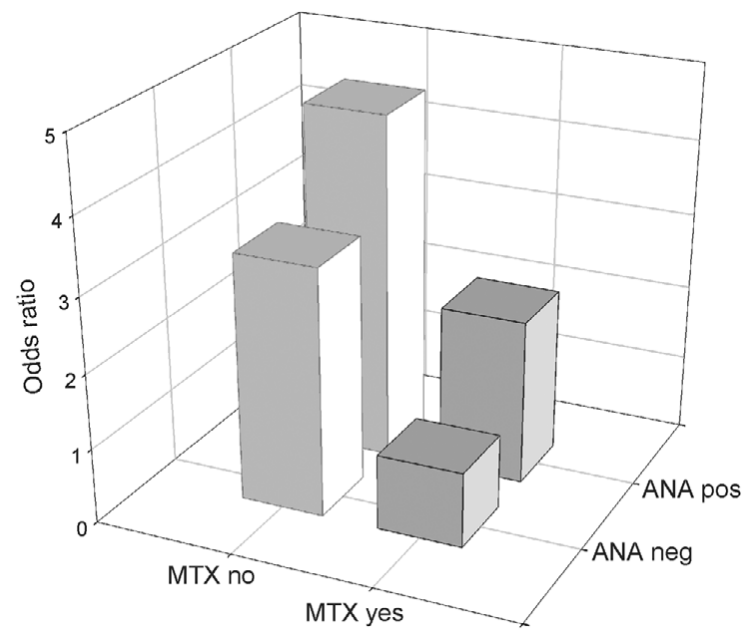
Positive predictive value for combination of baseline ANA status and methotrexate treatment for development of infusion reactions in RA patients. Values are expressed as Odds ratio adjusted for age, sex and disease duration. ANA, antinuclear antibody; MTX, methotrexate.

When applying the binary regression model, the presence of ANAs at treatment start and infliximab given without methotrexate or as monotherapy were each identified as independent risk factors for infusion reaction in patients with RA. Furthermore, the combination of both predictors was associated with further increased risk for developing an infusion reaction. ANA positivity at baseline and infliximab given without methotrexate were associated with the most pronounced risk. Consequently, RA patients without ANA positivity at treatment initiation and who were receiving infliximab in combination with methotrexate were least likely to develop an infusion reaction (Table 3 and Figure 1).

Concerning DMARDs other than methotrexate, the use of sulphasalazine, azathioprine and all other DMARDs as a group were not found to be associated with infusion reactions in the regression model. Lower age at disease onset and longer disease duration were associated with infusion reactions (*P *= 0.012 and *P *= 0.036, respectively), whereas age, sex, C-reactive protein, erythrocyte sedimentation rate, Health Assessment Questionairre and Disease Activity Score-28 at baseline did not influence this risk in patients with RA. No predictors of infusions reactions could be identified in SpA patients.

The stratification of infusion reactions according to grade of seriousness is shown in Table 4. The majority of patients with RA who developed serious or life-threatening reactions had clinical symptoms suggesting type I allergic reactions (anaphylactic: urticaria, hypotension, tachycardia, obstructive lung symptoms). These reactions led to withdrawal of infliximab treatment. However, a substantial proportion of the RA patients who developed infusion reactions had reactions that clinically were not judged as being allergic. Infusion reactions classified as moderate were mostly characterized by nonspecific symptoms, including headache, nausea, dizziness, fever or chills, chest or back pain, and coughing or general discomfort. These did not necessarily lead to discontinuation of the treatment. Mild infusion reaction symptoms were typically transient headache, fatigue and pain in general, and the majority of these patients could continue with treatment. Concerning SpA patients, three developed infusion reactions suggestive of type I allergic reactions. These reactions led to withdrawal of infliximab. Other infusion reactions were characterized by more nonspecific symptoms.

## Discussion

The main findings in the present study are that positive ANA status before the initiation of treatment with infliximab and use of infliximab without methotrexate in patients with RA are independent risk factors for developing infusion reactions; and that the risk is considerably increased in patients with a combination of both factors. The risk is most pronounced in ANA-positive patients treated with infliximab as monotherapy, suggesting that concomitant treatment with DMARDs, preferably methotrexate, should be encouraged before initiation of infliximab in RA patients. Concerning DMARDs other than methotrexate, use of sulphasalazine, of azathioprine and of all other DMARDs as a group were not associated with infusion reactions.

The association between new appearances of ANAs during infliximab treatment and the clinical consequences of these have been addressed in several studies [7-11]. However, to our knowledge, the present study is the first to report the predictive value of baseline ANA status for development of infusion reactions. ANA status is usually known or can be determined before the initiation of biologic treatments. Our results suggest that the presence of ANAs should be taken into account when infliximab treatment is considered.

The observation that combined treatment with infliximab and methotrexate was associated with reduced induction of anti-infliximab antibodies raised the hypothesis that concomitant methotrexate treatment reduces immunogenicity against monoclonal antibodies [2]. Also, patients with Crohn's disease experienced less frequent infusion reactions if infliximab treatment was given in combination with other immunosuppressive agents [6]. Our findings are in accordance with those reports and suggest that continuous methotrexate treatment should be encouraged in RA patients treated with inflximab and probably other monoclonal antibodies as well.

We found no association between baseline ANA status and use methotrexate and subsequent development of infliximab-related infusion reactions in patients with SpA. However, this finding must be interpreted with caution because of limited statistical power. In our study, concomitant treatment with methotrexate was used to a lesser extent in patients with SpA than in patients with RA, probably because methotrexate is not a prerequisite therapy in SpA patients. Furthermore, more frequent use of infliximab as monotherapy in SpA (48.9% versus 21.6%; Table 3) could be one possible explanation for the significant shorter duration of treatment at the moment of infusion reaction (4.3 months and 11.5 months for SpA and RA, respectively).

Additionally, more RA than SpA patients developed infusion reactions during the treatment. However, no significant difference in frequency of infusion reactions was observed between RA and SpA patients, possibly also reflecting the problem of statistical power. Provided that the same underlying mechanism is responsible for development of infusion reactions in both RA and SpA, the use of infliximab as monotherapy in a large proportion of SpA patients might have contributed to the nonsignificant difference.

In case of insufficient clinical response after 3 months, infliximab dosage could be increased. In a substantial proportion of both RA and SpA patients, infliximab dosage was increased over time but no clear association between increased dosage and infusion reactions could be detected.

A further interesting observation in the study is the unexpected high frequency (25%) of ANA positivity at baseline in SpA patients. In a recently reported review article by De Rycke and coworkers [9], which includes an overview of the studies investigating autoantibody profile during treatment with infliximab, ANAs were detected in between 4% and 17% of SpA patients before initiation of treatment. One possible explanation might be the use of different methods to detect ANAs. An advantage of international guidelines for clinical use of immunofluorescence assay for determination of ANAs is that comparisons of the results from different studies should be possible [17]. The method applied in our study is accredited and used routinely at Lund University Hospital. The prevalence of ANAs in our RA patients (27.9%) is comparable with that reported in the literature [9].

The mechanism underlying the association between ANA positivity and infusion reactions remains unknown. Immunological mechanisms are thought to be responsible for many of the toxic reactions to some DMARDs [12-14]. Furthermore, Panayi and coworkers found a positive correlation between HLA-DR phenotype and toxic complications of some DMARDs [14]. The findings of increased risk for developing infusion reactions in ANA-positive RA patients in our study support the plausibility of underlying immunogenetic mechanisms of drug-related side effects.

One weakness of this work is that determination of anti-infliximab antibodies was not performed. In a previously reported study including infliximab-treated patients with Crohn's disease, development of anti-infliximab antibodies of IgG type was found to be associated with increased risk for infusion reactions [6]. Our pilot study of development of anti-infliximab antibodies and infusions reactions in selected patients with RA [21] showed that antidrug antibodies were mostly of IgG and not of IgE type, despite clinical symptoms indicating type I allergic reactions. The clinical relevance of measuring these antibodies is currently being investigated.

In summary, RA patients in whom ANA positive status is present at treatment initiation with infliximab and who are treated without methotrexate are at increased risk for developing infusion reactions. The possible protective effects of DMARDs other than methotrexate against such infusion reactions remain to be studied.

## Conclusion

RA patients treated with infliximab without methotrexate and with positive baseline ANA status are at increased risk for developing infliximab-related infusion reactions. Both positive ANA status at baseline and non-use of concomitant methotrexate contributed to the development of infusion reactions in infliximab-treated RA patients.

## Competing interests

The authors declare that they have no competing interests.

## Authors' contributions

MCK was responsible for data analysis and interpretation, and wrote the manuscript. LL contributed to the collection of data, its interpretation and preparation of the manuscript. GS contributed to the idea and to the critical revision of the article. LT was responsible for laboratory analysis. TS contributed to critical revision of the manuscript and supervised the study. PG was responsible for the planning of the study and contributed to data analysis, data interpretation and preparation of the manuscript, and supervised the study. All authors read and approved the final manuscript.

## Figures and Tables

**Table 1:** Demographic characteristics of the patients, ANA status at baseline and characteristics of the infusion reactions

Characteristic	RA (*n *= 213)	SpAs (*n *= 76)
Age at inclusion (years)	55.9 ± 14.0	45.0 ± 13.1
Age at disease onset (years)	43.2 ± 15.2	31.9 ± 13.1
Disease duration at start (years)	12.6 ± 10.0	13.1 ± 11.0
Treatment duration at infusion reaction (months)	11.5 ± 9.6	4.3 ± 4.3
Number of previous DMARDs	3.3 ± 1.7	1.9 ± 1.1
Female	156 (73.2%)	40 (52.6%)
ANA positivity (yes)	56/201 (27.9%)	18/72 (25.0%)
Infusion reaction (yes)	45 (21.1%)	10 (13.2%)
Infusion reaction leading to withdrawal of treatment	33/45 (73.3 %)	9/10 (90%)

Values are expressed as mean ± standard deviation or as number (%). ANA, antinuclear antibody; DMARD, disease-modifying antirheumatic drug.

**Table 2:** Treatment characteristics and disease activity measures at baseline and at the infusion reaction in patients with RA and SpAs

Number	At treatment initiation		At infusion reaction	
	
	RA (*n *= 213)	SpA (*n *= 76)	RA (*n *= 45)	SpA (*n *= 10)
Drug treatments				
Infliximab monotherapy	46 (21.6%)	31 (40.8%)	22 (48.9%)	7 (70%)
Methotrexate	129 (60.6%)	35 (46.1%)	17 (37.8%)	2 (20%)
Sulphasalazine	33 (15.5%)	6 (7.9%)	5 (11.1%)	0
Azathioprine	10 (4.7%)	3 (3.9%)	1 (2.2%)	0
Other DMARDs	30 (14.1%)	4 (5.3%)	4 (8.9%)	1 (10%)
Prednisolone	155 (72.8%)	30 (39.5%)	38 (84.4)	6 (60%)
Dosages				
Methotrexate (mg/week)	17.3 ± 5.2	15.6 ± 6.8	15.4 ± 6.1	16.2 ± 12.4
Sulphasalazine (g/week)	13.5 ± 2.3	15.2 ± 2.9	15.4 ± 3.1	None
Azathioprine (mg/week)	745.5 ± 432.1	758.3 ± 101.0	700	None
Prednisolone (mg/week)	43.2 ± 37.8	20.5 ± 28.9	33.4 ± 29.0	11.7 ± 18.0
Disease activity measures				
DAS-28	5.4 ± 1.3	-	5.0 ± 1.6	-
HAQ	1.4 ± 0.6	1.1 ± 0.6	1.3 ± 0.6	1.1 ± 0.8
CRP	30.9 ± 33.4	21.7 ± 26.2	25.5 ± 26.3	14.4 ± 16.9
ESR	36.8 ± 27.2	27.4 ± 23.0	37.8 ± 25.5	29.2 ± 32.1
VASpain (0–100)	61.7 ± 22.2	61.4 ± 19.7	48.3 ± 25.7	55.9 ± 32.3
VASglobal (0–100)	63.4 ± 21.6	63.4 ± 19.7	52.5 ± 25.4	56.3 ± 33.6
Evaglobal (0–5)	2.3 ± 0.6	1.8 ± 0.7	1.9 ± 0.9	1.8 ± 0.6
TJC (0–28)	8.7 ± 7.3	5.9 ± 6.9	7.9 ± 7.8	6.6 ± 8.3
SJC (0–28)	8.9 ± 6.0	3.0 ± 4.1	6.9 ± 6.0	3.3 ± 5.4

Values are expressed as mean ± standard deviation or as number (%). CRP, C-reactive protein; DAS-28, Disease Activity Score (using 28 tender and 28 swollen joint count); DMARD, disease-modifying antirheumatic drug; ESR, erythrocyte sedimentation rate; Evaglobal, physicians global assessment; HAQ, Health Assessment Questionnaire; RA, rheumatoid arthritis; SJC, swollen joint count; SpA, spondylarthropathy; TJC, tender joint count; VASpain, patient's assessment of pain; VASglobal, patient's global assessment.

**Table 3:** Positive predictive values for separate factors and combination of presence of ANAs and methotrexate treatment for development of infusion reactions in RA patients

Factors	Patients (*n*)	OR	95% CI	*P*
ANA positivity	56	2.1	1.04–4.29	0.040^a^
Infliximab without methotrexate	84	3.1	1.53–6.29	0.002^a^
Infliximab as monotherapy	46	3.6	1.73–7.14	0.001^a^
				
ANA positive + MTX no	26	4.6	1.61–13.15	0.004^b^
ANA positive + MTX yes	30	2.2	0.74–6.36	0.161^b^
ANA negative + MTX yes	93	1.0	-	-
ANA negative + MTX no	52	3.3	1.35–8.06	0.009^b^

^a^Adjusted for age, sex and prednisolone at start. ^b^Adjusted for age, sex and disease duration. ANA, antinuclear antibody; CI, confidence interval; MTX, methotrexate; OR, odds ratio; RA, rheumatoid arthritis.

**Table 4:** Number of infusion reactions classified according to grade of seriousness

Group	Grade of seriousness	Infusion reactions				
		
		All	Infliximab dosage unchanged	Infliximab dosage increased at 3–6 months	Infliximab dosage increased at 6–12 months	Infliximab dosage increased at 12 months
RA patients (*n *= 45)	Life threatening	2	1	1	0	0
	Serious	16	11	2	1	2
	Moderate	24	15	8	5	6
	Mild	3	1	0	1	1
SpA patients (*n *= 10)	Life threatening	0	0	0	0	0
	Serious	6	4	1	1	0
	Moderate	2	1	1	0	0
	Mild	2	1	1	1	0

RA, rheumatoid arthritis; SpA, spondylarthropathy.

## References

[B1] Maini R, St Clair EW, Breedveld F, Furst D, Kalden J, Weisman M, Smolen J, Emery P, Harriman G, Feldmann M (1999). Infliximab (chimeric anti-tumour necrosis factor alpha monoclonal antibody) versus placebo in rheumatoid arthritis patients receiving concomitant methotrexate: a randomised phase III trial. Lancet.

[B2] Maini RN, Breedveld FC, Kalden JR, Smolen JS, Davis D, Macfarlane JD (1998). Therapeutic efficacy of multiple intravenous infusions of anti-tumor necrosis factor alpha monoclonal antibody combined with low-dose weekly methotrexate in rheumatoid arthritis. Arthritis Rheum.

[B3] Braun J, Brandt J, Listing J, Zink A, Alten R, Golder W, Gromnica-Ihle E, Kellner H, Krause A, Schneider M (2002). Treatment of active ankylosing spondylitis with infliximab: a randomised controlled multicentre trial. Lancet.

[B4] Brandt J, Braun J (2006). Anti-TNF-alpha agents in the treatment of psoriatic arthritis. Expert Opin Biol Ther.

[B5] Cheifetz A, Mayer L (2005). Monoclonal antibodies; immunogenicity; and associated infusion reactions. Mt Sinai J Med.

[B6] Baert F, Noman M, Vermeire S, Assche GV, Haens GD, Carbonez A, Rutgeerts P (2003). Influence of immunogenicity on the long-term efficacy of infliximab in Crohn's disease. N Engl J Med.

[B7] De Rycke L, Kruithof E, Van Damme N, Hoffman IE, Van den Bossche N, Van den Bosch F, Veys EM, De Keyser F (2003). Antinuclear antibodies following infliximab treatment in patients with rheumatoid arthritis or spondylarthropathy. Arthritis Rheum.

[B8] Elkayam O, Burke M, Vardinon N, Zakut V, Yitzhak RB, Paran D, Levartovsky D, Litinsky I, Caspi D (2005). Autoantibodies profile of rheumatoid arthritis patients during treatment with infliximab. Autoimmunity.

[B9] Rycke LD, Baeten D, Kruithof E, Bosch FV, Veys EM, Keyser FD (2005). The effect of TNF alpha blockade on the antinuclear antibody profile in patients with chronic arthritis: biological and clinical implications. Lupus.

[B10] Eriksson C, Engstrand S, Sundqvist KG, Rantapaa-Dahlqvist S (2005). Autoantibody formation in patients with rheumatoid arthritis treated with anti-TNF alpha. Ann Rheum Dis.

[B11] Paulus HE, Wiesner J, Bulpitt KJ, Patnaik M, Law J, Park GS, Wong WK (2002). Autoantibodies in early seropositive rheumatoid arthritis; before and during disease modifying antirheumatic drug treatment. J Rheumatol.

[B12] Ferraccioli GF, Nervetti A, Mercadanti M, Cavaliri F (1986). Serum IgA levels and ANA behaviour in rheumatoid patients with and without toxicity to remission-inducing drugs. Clin Exp Rheumatol.

[B13] Schmidt KL, Mueller-Eckhardt C, Breithaupt H (1978). HLA-B27; antinuclear antibodies and drug-induced agranulocytosis. Klin Wochenschr.

[B14] Panayi GS, Wooley P, Batchelor JR (1978). Genetic basis of rheumatoid disease: HLA antigens; disease manifestations; and toxic reactions to drugs. Br Med J.

[B15] Arnett FC, Edworthy SM, Bloch DA, McShane DJ, Fries JF, Cooper NS, Healey LA, Kaplan SR, Liang MH, Luthra HS (1988). The American Rheumatism Association 1987 revised criteria for the classification of rheumatoid arthritis. Arthritis Rheum.

[B16] der Linden S, Valkenburg HA, Cats A (1984). Evaluation of diagnostic criteria for ankylosing spondylitis. A proposal for modification of the New York criteria. Arthritis Rheum.

[B17] Moll JMH, Wright V (1973). Psoriatic arthritis. Semin Arthritis Rheum.

[B18] Geborek P, Crnkic M, Petersson IF, Saxne T (2002). Etanercept, infliximab and leflunomide in established rheumatoid arthritis: clinical experience using a structured follow up programme in southern Sweden. Ann Rheum Dis.

[B19] Fransen J, van Riel PL (2005). The Disease Activity Score and the EULAR response criteria. Clin Exp Rheumatol.

[B20] Kavanaugh A, Tomar R, Reveille J, Solomon DH, Homburger AH (2000). Guidelines for clinical use of the antinuclear antibody test and tests for specific autoantibodies to nuclear antigens. American College of Pathologists. Arch Pathol Lab Med.

[B21] Kapetanovic MC, Geborek P, Saxne T, Larsson L, Kristensen L, Svenson M, Bendtzen K (2005). Development of antibodies against infliximab during infliximab treatment in rheumatoid arthritis. Relation to infusion reactions and treatment response [abstract 1440]. Arthritis Rheum.

